# Toward Clinically Feasible Assessment of Muscle Mass: Validation of a Seated Bioelectrical Impedance Device Against Dual-Energy X-Ray Absorptiometry (DXA) in Older Adults

**DOI:** 10.3390/s26165112

**Published:** 2026-08-12

**Authors:** Elodie Piche, Emeline Michel, Olivier Guerin, Etienne Gouraud, Frédéric Chorin

**Affiliations:** 1 Laboratoire Motricité Humaine Éducation Sport Santé (LAMHESS), Université Côte d’Azur, 06100 Nice, Francechorin.f@chu-nice.fr (F.C.); 2 Clinique Gériatrique du Cerveau et du Mouvement, Centre Hospitalier Universitaire de Nice, 06100 Nice, France; 3 Institute for Research on Cancer and Aging Nice (IRCAN), CNRS UMR 7284/INSERM U108, Faculté de Médecine, Université Côte d’Azur, 06100 Nice, France; 4 Aminogram SAS, 13600 La Ciotat, France

**Keywords:** sarcopenia, bioelectrical impedance analysis, dual-energy X-ray absorptiometry, appendicular skeletal muscle mass, phase angle, older adults, muscle mass assessment

## Abstract

**Highlights:**

**What are the main findings?**

- A seated bioelectrical impedance sensor accurately estimated appendicular muscle mass against DXA in older adults.
- The Sergi prediction equation provided higher agreement and lower estimation error than the Kyle equation.

**What are the implications of the main findings?**

- Seated bioelectrical impedance enables rapid, practical muscle mass assessment without compromising measurement validity.
- The phase angle derived from the sensor provides complementary information on muscle function and sarcopenia.

**Abstract:**

**Background**: Although dual-energy X-ray absorptiometry (DXA) is the reference method for measuring appendicular skeletal muscle mass (ASMM) and diagnosing sarcopenia, seated bioelectrical impedance analysis (BIA) may represent a practical alternative. As its validity remains still insufficiently documented in specific populations, the objective is to evaluate the agreement between seated BIA and DXA, and examine associations between phase angle (PhA) and physical performance in old adults. **Methods**: Fifty-nine old adults (75 ± 7 years) underwent DXA and seated BIA (Biody XpertZM3). ASMM was estimated using the Kyle and Sergi equations. Agreement with DXA was assessed using Lin’s concordance correlation coefficient (CCC), Bland–Altman analysis, root mean square error (RMSE) and mean absolute error (MAE). Associations between PhA, handgrip strength, and five-times sit-to-stand (5-STS) performance were evaluated. **Results**: Both equations showed good agreement with DXA, but the Sergi equation performed better, with higher concordance (CCC = 0.942 vs. 0.898), lower bias (0.44 vs. 1.11 kg), and lower RMSE (1.33 vs. 1.96 kg). PhA correlated positively with handgrip strength (r = 0.315, *p* = 0.015) but was not associated with 5-STS. **Conclusions**: Seated BIA provides clinically acceptable estimates of ASMM in older adults. The Sergi equation demonstrated superior accuracy, supporting population-specific equations. PhA may provide additional information on muscle quality and sarcopenia.

## 1. Introduction

Sarcopenia is defined as a progressive and generalized loss of muscle strength and mass associated with aging [1]. Combined with reduced physical activity and inadequate nutritional intake, it contributes to the development of frailty, negatively impacting quality of life, health status, and the ability to age independently and successfully [2]. Currently, the prevention and management of sarcopenia primarily rely on the combination of regular physical activity and adequate protein intake, with the aim of preserving muscle function and mass [3,4].

Since its introduction by Irwin H. Rosenberg in 1988 [5], the concept of sarcopenia has evolved considerably. The revised definition proposed by the European Working Group on Sarcopenia in Older People (EWGSOP2, 2019) uses low muscle strength as the primary criterion, while reduced muscle mass serves as a confirmatory criterion [1]. The French National Authority for Health (HAS) has adopted these recommendations for the screening of sarcopenia and malnutrition in older adults during routine clinical practice [6].

In the context of an aging population, early identification of sarcopenia has become a major clinical challenge. Accurate assessment of appendicular skeletal muscle mass (ASMM) is therefore essential. Current guidelines recommend dual-energy X-ray absorptiometry (DXA) and bioelectrical impedance analysis (BIA) for this purpose [6]. While DXA is considered as the reference method, its use is limited by high cost, limited accessibility, and lack of portability. BIA represents a more practical alternative due to its lower cost and ease of use. It estimates body composition based on the electrical properties of tissues, using predictive equations to derive parameters such as fat-free mass and ASMM [7]. 

Several BIA predictive equations have already been developed and validated in older populations [8,9], including those proposed by Kyle and Sergi [10,11]. However, previous validation have consistently shown that the accuracy of BIA-derived ASMM strongly depends on the prediction equation used, with important variations according to ethnicity [12], age [10,13], sex [13], and the population in which the equation was developed. Consequently, equations validated in one population may not be extrapolated automatically to another [13,14,15]. 

Conventional BIA measurements are also subject to several sources of variability, including hydration status, temperature, electrode placement and, notably, body position during assessment [15,16]. To reduce this variability, standardized protocols recommend measurements in a supine position after a resting period, with electrodes placed on the hand and foot [17]. Body position is particularly relevant because postural changes redistribute body fluids between the trunk and the extremities, thereby altering the electrical pathways measured by BIA [18]. Studies comparing measurements obtained in lying, sitting, and standing positions have reported significant differences in resistance, reactance, phase angle, and water-related indices [18,19]. In addition, bioelectrical measurements may continue to change during the first minutes after assuming a supine position, reflecting the progressive redistribution of body fluids and supporting the need for a standardized resting period [20]. Although these conditions improve measurement reliability, they may also introduce practical constraints, particularly in frail older adults, for whom maintaining a supine position and adhering to standardized preparation procedures may be less convenient in some clinical settings.

In this context, the development of alternative approaches that maintain measurement reliability while improving feasibility is of particular interest. Seated-position BIA devices with integrated electrodes offer a promising solution, as they allow rapid, non-invasive assessment without requiring the patient to lie down or undergo complex preparation. Thus, this posture may be more consistent with routine clinical practice. Moreover, previous studies have shown good agreement between such devices and reference methods, including DXA and standard BIA systems used to derive predictive equations [21], although validation in older populations remains limited. Despite these promising findings, evidence regarding the validity of seated BIA devices equipped with integrated electrodes remains scarce in older adults, in whom age-related changes in body composition, hydration status, and sarcopenia may influence impedance-derived estimates. Furthermore, because the accuracy of BIA depends not only on the sensor itself but also on the prediction equation used, the performance of commonly used equations has not been sufficiently investigated in this novel measurement configuration. Therefore, validating both the sensing technology and the associated prediction models is essential before such devices can be implemented in routine geriatric assessment. Importantly, given that non-standardized body positions can introduce significant variability in BIA-derived estimates [22], validation of measurements performed in a seated position is particularly warranted. 

Beyond muscle mass estimation, bioelectrical impedance also provides access to phase angle (PhA), a marker reflecting cellular integrity and hydration status, which has been associated with muscle function and clinical outcomes in older adults [23,24,25]. A recent systematic review suggests that PhA provides information related to muscle quality and shows moderate diagnostic performance for sarcopenia, although reported cutoffs and associations vary across populations [26]. However, body position may theoretically influence PhA values, highlighting the need to characterize its behavior in a seated position and to verify its relationship with muscle mass and strength in this context.

Therefore, the aim of the present study was to validate a novel seated bioelectrical impedance sensor equipped with integrated electrodes against the reference method DXA for estimating appendicular skeletal muscle mass in older adults. In addition, we compared the performance of two widely used prediction equations (Kyle and Sergi), evaluated whether sarcopenia influences measurement validity, and explored the clinical relevance of phase angle obtained from this sensor configuration. It is hypothesized that both the Kyle and Sergi predictive equations will provide valid estimates of ASMM when applied within the same BIA device. We also hypothesized that the validity of the BIA measurements will not be significantly influenced by the presence of sarcopenia. Finally, the study hypothesizes that PhA will be significantly associated with ASMM and handgrip strength, in line with previous findings [23,24], supporting the relevance of bioelectrical impedance measurements performed in a seated position.

## 2. Materials and Methods

### 2.1. Participants

Fifty-nine adults aged over 60 years were recruited for this study. All participants underwent a comprehensive clinical evaluation as part of their routine consultation. The study was registered (n°ID-RCB: 2025-A00799-40) and approved by the *Comité de Protection des Personnes Sud-Est II*. The study was also conducted in accordance with the principles of the Declaration of Helsinki. All participants provided written informed consent prior to participation. 

### 2.2. Classification of Sarcopenia

Participants were classified into groups according to the presence or absence of sarcopenia, based on the diagnostic criteria established by the European Working Group on Sarcopenia in Older People (EWGSOP2, 2019).

Initial screening for sarcopenia was conducted using the SARC-F questionnaire, a validated five-item screening tool. A score ≥4 was considered suggestive of sarcopenia and prompted further assessment. Muscle strength was assessed using handgrip strength and the chair stand test, while muscle mass was measured using BIA and DXA. According to the EWGSOP2 criteria, participants presenting low muscle strength (handgrip strength and/or chair stand test below the recommended thresholds) together with low ASMM were classified as sarcopenic (Table 1). Participants who did not fulfill both criteria were classified as non-sarcopenic.

### 2.3. Assessments and Measurements

After the medical consultation, all participants underwent a series of physical and body composition assessments. Handgrip strength was measured using a handheld dynamometer (Handgrip, Kinvent, Biomécanique SAS, Montpellier, France). Participants performed maximal voluntary contractions lasting 5 seconds, with two trials conducted for each hand. The highest value obtained was retained for analysis. Lower limb function was assessed using the five-repetition sit-to-stand (5-STS) test. Participants were instructed to rise from a seated position and sit back down five times as quickly as possible, with their arms crossed over the chest. The time required to complete the test was recorded. Two trials were performed, and the best performance was retained for analysis. The test was conducted using a standard chair with a seat height of 45 cm.

Then, body composition was assessed using dual-energy X-ray absorptiometry (DXA) with the STRATOS dR densitometer (DMS Imaging, Mauguio, France). DXA is considered the reference method for body composition assessment because it uses two low-energy X-ray beams to differentiate bone mineral content, lean soft tissue, and fat mass, providing highly accurate and reproducible estimates of appendicular skeletal muscle mass (ASMM). Although associated with very low radiation exposure, its routine clinical use is limited by the need for specialized equipment, dedicated facilities, trained personnel, and relatively high costs. DXA-derived Appendicular Skeletal Muscle Mass (ASMM DEXA, in kg) and Appendicular Skeletal Muscle Mass Index (ASMI DEXA, in kg/m^2^) were used as the reference measures in the present study. In addition, BIA was performed using the Biody XpertZM 3 device (AMINOGRAM SAS, La Ciotat, France). This non-invasive multifrequency bioelectrical impedance analysis system is equipped with four integrated electrodes and allows measurements to be conducted in a seated position, with electrodes placed on the fingers and the heel, just below the malleolus, enabling tetrapolar whole-body impedance measurements between the upper and lower limbs. Before each measurement, participants were instructed to place their hands and feet directly on the electrodes according to the manufacturer’s recommendations to ensure consistent electrode–skin contact. This seated measurement protocol eliminates the need for adhesive electrodes, thereby reducing consumables and improving ease of use for both the clinician and the participant. However, the change in posture from supine to seated, as well as the modification in electrode placement, may influence bioelectrical measurements and, consequently, the estimation of appendicular lean mass. During measurement, a current of 0.035 mA delivered at 50 kHz is applied through the electrodes and travels between the hand and foot. This current is well below the sensory threshold of the nervous system [27], making the procedure painless and safe. ASMM and ASMI were estimated using the seated BIA device according to two predictive equations implemented within the manufacturer’s software: the Kyle equation and the Sergi equation. The Kyle equation was originally developed into a heterogeneous adult population [10], whereas the Sergi equation was specifically derived and validated in older adults using DXA as the reference method [11]. Because age-related changes in body composition and hydration status may influence the accuracy of BIA-derived muscle mass estimates, both equations were evaluated in the present study to determine their agreement with DXA-derived ASMM and ASMI and to identify the most appropriate prediction model for use in older adults. The order of the two body composition assessments (DXA and BIA) was randomized.

### 2.4. Statistical Analysis

Statistical analyses were performed using JASP software (v. 0.19.1.0; JASP Team, University of Amsterdam, The Netherlands). Data distribution was assessed using the Shapiro–Wilk test and visual inspection of histograms and Q–Q plots. Continuous variables were expressed as mean ± standard deviation (SD) for normally distributed data or as median and interquartile range (IQR) when appropriate, while categorical variables were presented as frequencies and percentages. Statistical significance was set at *p* < 0.05.

Agreement between appendicular skeletal muscle mass (ASMM) measured by DXA and estimates derived from the seated-position BiodyXpert device using the Kyle and Sergi predictive equations was assessed using complementary statistical approaches. Agreement was quantified using Lin’s concordance correlation coefficient (CCC), which evaluates both precision and accuracy relative to the line of identity. Systematic bias was assessed using Bland–Altman analyses, reporting mean bias and 95% limits of agreement (LoA). Proportional bias was evaluated using linear regression between the differences (BIA – DXA) and the mean of both methods. Mean absolute error (MAE) and root mean square error (RMSE) were calculated to quantify absolute and quadratic prediction errors relative to DXA.

To determine whether sarcopenia influenced the validity of BIA-derived measurements, estimation error was calculated for each equation as the difference between BIA-derived ASMM and DXA-derived ASMM (BIA – DXA). Multiple linear regression analyses were then performed to assess whether sarcopenia status independently predicted estimation error after adjustment for potential confounders, including age, sex, and body mass index (BMI). Separate models were conducted for the Kyle and Sergi predictive equations. Standardized regression coefficients, 95% confidence intervals, and model fit indices (R^2^) were reported. In addition, subgroup agreement analyses using ICC and Bland–Altman plots were performed separately in sarcopenic and non-sarcopenic participants to further examine potential differences in measurement validity.

Associations between PhA, 5-STS time, and handgrip strength were assessed using Pearson’s correlation coefficients, or Spearman’s rank correlations when normality assumptions were not met. 

## 3. Results

### 3.1. Clinical and Demographical Descriptions

Fifty-nine participants were included in the study, with a mean age of approximately 75 years (range: 60.37–94.29 years). As expected, participants classified as sarcopenic exhibited higher SARC-F scores, lower handgrip strength, and longer 5-STS completion times than non-sarcopenic participants. In addition, the sarcopenic group was significantly older than the non-sarcopenic group (Table 2). Height, weight and handgrip force were different between males and females (Table 2). 

Concerning the muscle mass measures, ASMM measured by DXA was 16.84 ± 3.87 kg. Using the BIODY XpertZM3, ASMM was 17.95 ± 4.69 kg when calculated with the Kyle equation, and 17.28 ± 3.97 kg when calculated with the Sergi equation (Table 2). No significant differences were highlighted in any of the muscle mass values between the non-sarcopenic and sarcopenic group (*p* > 0.05, Table 2). However, between male and female, ASMM measured with the DEXA or the BIODY XpertZM3 using Kyle or Sergi equation was significantly different (Table 2). 

Concerning PhA, the value was significantly different between sarcopenic and non-sarcopenic participants (*p* < 0.001, Table 2) but not between male and female participants (*p* > 0.05). 

### 3.2. Agreement Between DXA and BiodyXpert for ASMM and ASMI

**Figure 1:**
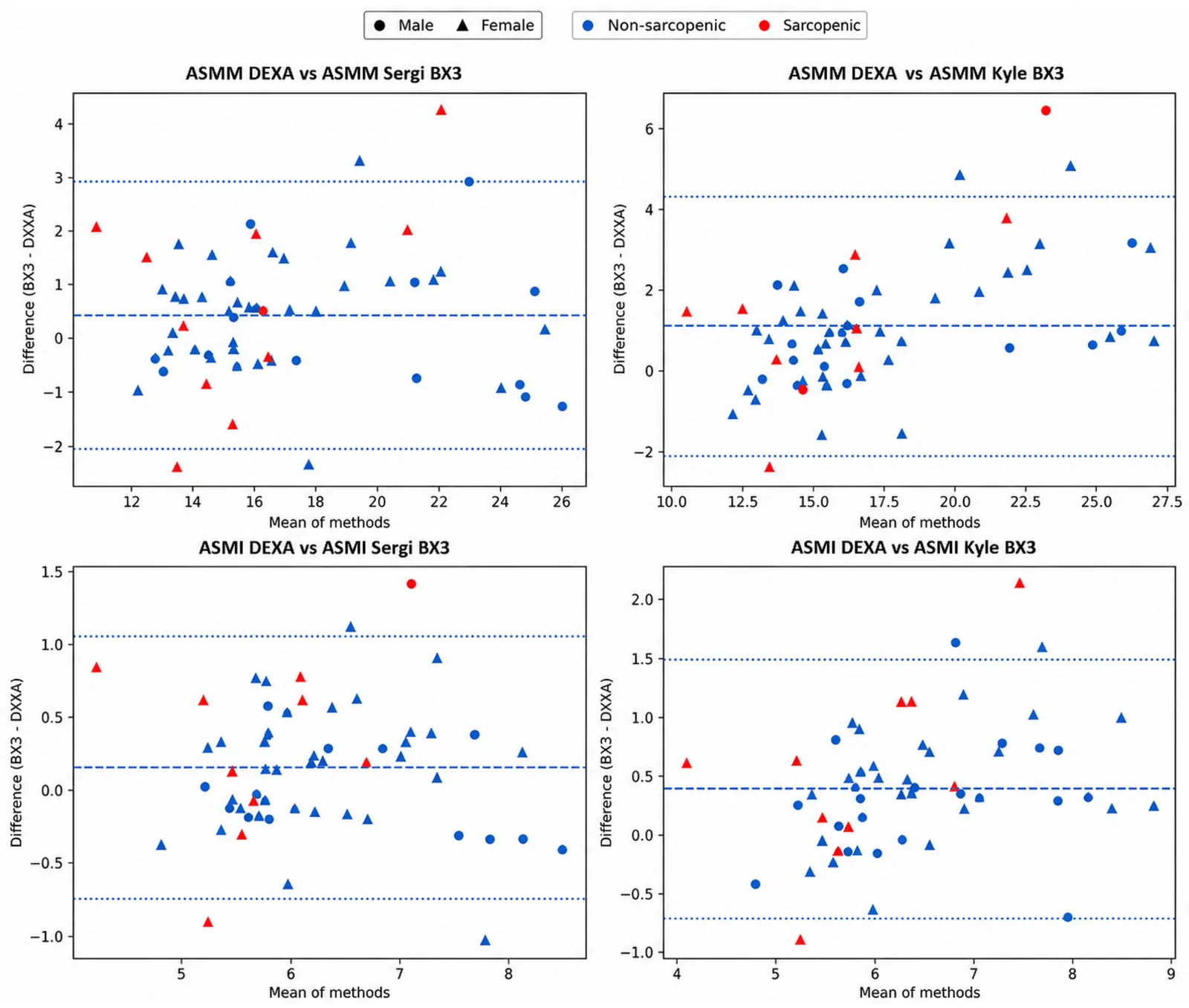
Bland–Altman plots showing the agreement between appendicular skeletal muscle mass (ASMM) and appendicular skeletal muscle mass index (ASMI) measured by dual-energy X-ray absorptiometry (DXA) and estimated using the seated bioelectrical impedance analysis (BIA) device with the Sergi and Kyle equations. Red symbols represent sarcopenic participants and blue symbols represent non-sarcopenic participants. Circles indicate males and triangles indicate females. The blue dotted line represent the mean difference (bias) between the two measurement methods, while the two thin dotted lines represent the represent the limits of agreement (mean ± 1.96 * standard deviation of the differences).

Bland–Altman analyses demonstrated that the Sergi-corrected BiodyXpert estimates were consistently in closer agreement with DXA than the Kyle-corrected estimates. For ASMM, the Sergi equation showed a mean bias of 0.44 kg with 95% limits of agreement (LoA) from −2.04 to 2.92 kg, whereas the Kyle equation showed a larger bias of 1.11 kg and wider LoA (−2.08 to 4.30 kg) (Figure 1, Table 2). Similarly, for ASMI, the Sergi equation exhibited a lower mean bias (0.16 kg/m^2^; 95% LoA: −0.74 to 1.05 kg/m^2^) compared with the Kyle equation (0.39 kg/m^2^; 95% LoA: −0.71 to 1.48 kg/m^2^) (Figure 1, Table 3). Regression analyses of Bland–Altman differences revealed no proportional bias for the Sergi equation for either ASMM (*p* = 0.517) or ASMI (*p* = 0.744), indicating stable agreement across the measurement range. In contrast, the Kyle equation demonstrated significant proportional bias for both ASMM (slope = 0.198, *p* < 0.001) and ASMI (slope = 0.190, *p* = 0.009), indicating increasing overestimation with higher muscle mass values (Table 3). Visual inspection of the plots showed a similar distribution of sarcopenic and non-sarcopenic participants across the range of measurements, suggesting that sarcopenia status did not materially affect the agreement between methods (Figure 1).

Concerning concordance analysis, for ASMM, the Sergi equation demonstrated higher concordance with DXA (CCC = 0.942) than the Kyle equation (CCC = 0.898) (Table 3). For ASMI, a similar pattern was observed, with higher concordance for the Sergi equation (CCC = 0.865) compared with the Kyle equation (CCC = 0.796) (Table 3). Visual inspection of the Bland–Altman plots suggested that agreement between DXA and BIA-derived estimates was not noticeably influenced by sex, as male and female participants were similarly distributed across the measurement range without an apparent difference in the magnitude of the measurement bias (Figure 1).

The results for the error-based metrics showed that for ASMM, both RMSE and MAE were lower for the Sergi equation (RMSE = 1.33 kg; MAE = 1.04 kg) than for the Kyle equation (RMSE = 1.96 kg; MAE = 1.46 kg) (Table 3). For ASMI, the Sergi equation again showed lower error (RMSE = 0.48 kg/m^2^; MAE = 0.38 kg/m^2^) compared with the Kyle equation (RMSE = 0.68 kg/m^2^; MAE = 0.53 kg/m^2^) (Table 3).

### 3.3. Correlation Between Phase Angle and Physical Performance (Handgrip and 5-STS)

**Figure 2:**
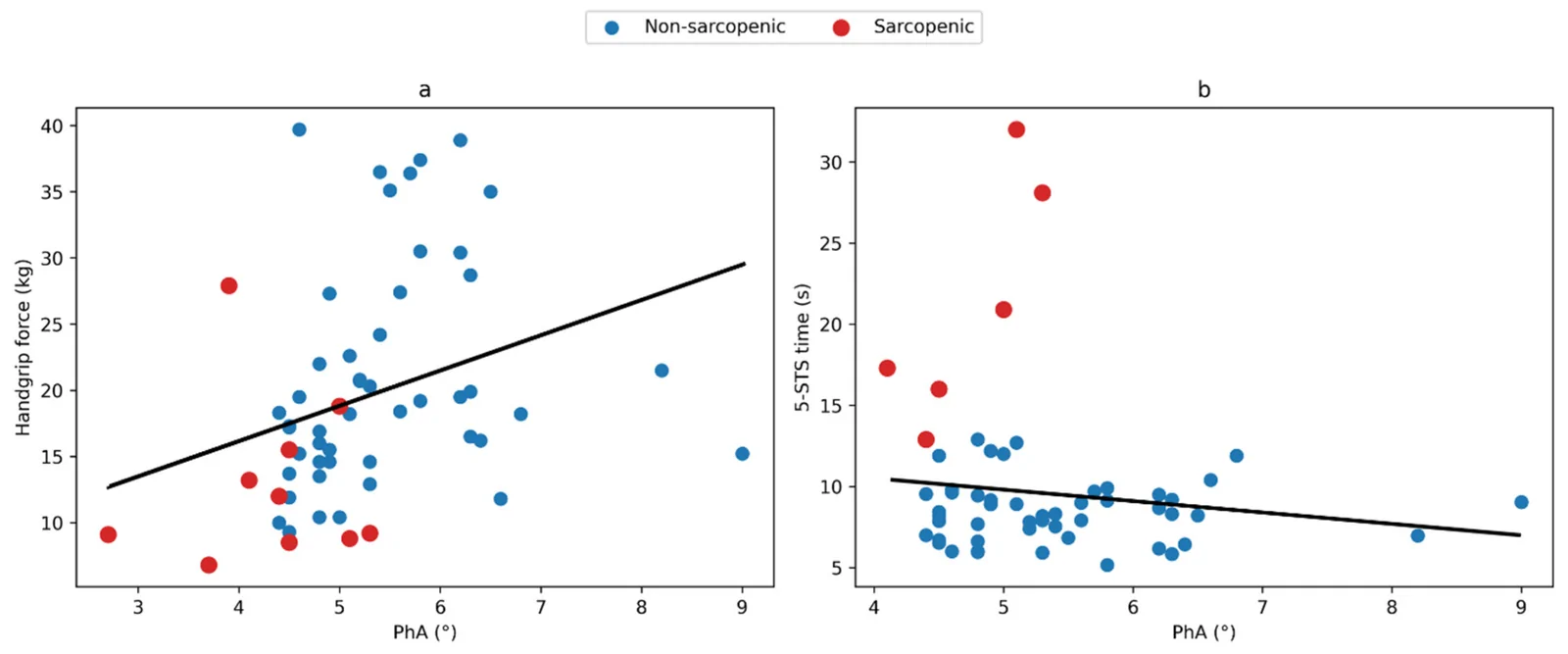
Relationship between phase angle (PhA) and physical performance measures. (**a**) Correlation between phase angle (PhA, °) and maximal handgrip force (kg). (**b**) Correlation between phase angle (PhA, °) and 5-times sit-to-stand (5-STS time (s)). Blue circles represent non-sarcopenic participants and red circles represent sarcopenic participants. The solid black line represents the linear regression fit.

Results highlighted a moderate positive correlation between PhA of the BIODY XpertZM3 and the maximal handgrip force (Figure 2a; r = 0.315, *p* = 0.015) while no significant correlation was shown between PhA and the 5-STS time (Figure 2b; r = −0.142, *p* = 0.300). It is important to note that 4 sarcopenic participants were not able to stand up from their chair so only 6 participants appeared in Figure 2b. 

## 4. Discussion

The present study evaluated the agreement between a seated-position BIA device and DXA for assessing ASMM and ASMI in older adults. We also compared the performance of the Kyle and Sergi predictive equations, assessed the influence of sarcopenia on measurement validity, and explored associations between PhA, muscle mass, and physical performance. Overall, both equations showed good agreement with DXA, but the Sergi equation performed better, with higher concordance, lower error, and no proportional bias. In contrast, the Kyle equation systematically overestimated ASMM and showed proportional bias, particularly at higher muscle mass values. PhA was positively associated with handgrip strength but not with 5-STS performance, supporting its relevance as a marker of muscle function and cellular health. These findings partially confirmed the hypotheses, with Sergi providing the most robust estimates and PhA showing meaningful functional associations.

Results indicate that the seated BIA device provides clinically acceptable estimates of ASMM and ASMI in older adults when compared with DXA, with agreement metrics comparable to those reported in previous validation studies of conventional BIA systems. However, the choice of predictive equation markedly influenced measurement accuracy. The Sergi equation demonstrated superior agreement with DXA, characterized by higher CCC, lower RMSE and MAE, reduced systematic bias, and, importantly, the absence of proportional bias. In contrast, the Kyle equation showed a consistent overestimation of ASMM and ASMI, with error increasing alongside higher muscle mass values. This pattern suggests a scaling issue in the underlying assumptions of the Kyle model when applied in older adults. These findings are consistent with previous studies showing that the accuracy of BIA-derived estimates of appendicular skeletal muscle mass depends strongly on the prediction equation used [22,27]. In particular, equations developed and validated in older adults appear to provide more accurate estimates in geriatric populations than equations derived from heterogeneous adult cohorts, likely because they had better account for age-related changes in body composition and hydration status [28]. Cañez-Ríos et al. [28] demonstrated that the agreement between BIA-derived and DXA-derived ASMM varied substantially according to the prediction equation applied, highlighting the importance of selecting equations that are appropriate for the target population. The observed discrepancies between the Kyle and Sergi equations are unlikely to be explained by the seated measurement condition, as both prediction models were derived from the same impedance measurements and therefore relied on identical resistance and reactance values. Instead, these differences probably reflect the populations and assumptions underlying each equation. The Kyle equation was developed using a mixed adult population and relies on assumptions regarding whole-body conductivity and segmental distribution of lean mass [10]. In contrast, the Sergi equation was specifically developed and validated in older adults and may therefore better capture age-related changes in body composition, including alterations in body geometry and hydration status [11,14]. Consequently, the systematic overestimation observed with the Kyle equation likely reflects the reduced suitability of its underlying assumptions when applied to geriatric populations. In this context, equations such as Sergi’s, which appear less sensitive to these variations, may provide more stable estimates in non-standardized clinical conditions. Our findings are consistent with previous validation studies reporting better performance of geriatric-specific equations in older adults and frail populations. Similarly, Vermeiren et al. [9] developed a population-specific equation for adults aged 80 years and older, further supporting the need to tailor BIA prediction equations to the characteristics of the population under investigation. Taken together, these results support the use of population- and device-specific calibration rather than a universal equation approach. Moreover, we found similar systematic bias and CCC with the Sergi equation in our study compared to previous publications [28,29], showing that there are no differences in performance between standardized supine position with self-adhesive electrodes and seated position with integrated electrodes in older adults. Consequently, our results support the use of seated BIA as a practical alternative for routine muscle mass assessment when DXA is unavailable or impractical, rather than as a replacement for the reference method.

PhA reflects the capacitive component of the cell membrane, i.e., its capacity to retain electrical charges, and is considered as an indirect marker of cellular integrity, membrane function, and body cell mass [30]. Higher values are generally associated with better cell membrane integrity and higher muscle quality, whereas lower values indicate cellular degradation, inflammation, or malnutrition [23]. In older adults, PhA has also been associated with appendicular skeletal muscle mass, body cell mass, and body fat percentage, supporting its use as a broader clinical and nutritional assessment parameter rather than as a marker of muscle mass alone [24]. In the present study, PhA showed a moderate positive correlation with handgrip strength, but not with the 5-STS test. This pattern is consistent with previous work showing that PhA is often associated with grip strength and other muscle-strength measures in older adults [31]. These findings suggest that PhA is more closely related to upper limb muscle strength than to functional lower limb performance in this cohort. This may reflect differences in neuromuscular recruitment patterns, test sensitivity, or the multifactorial nature of the sit-to-stand movement, which involves balance, coordination, and joint mobility in addition to muscle strength. Our results are in line with previous studies demonstrating the association between PhA and muscle strength, functional decline, and clinical outcomes in older adults [23,24,32]. For example, Basile et al. [23] reported that lower PhA values were associated with reduced upper limb strength while Grootswagers et al. [24] confirmed the link between PhA and several functional physical performance measures. Also, this study found that PhA was significantly different between sarcopenic and non-sarcopenic participants, with a significantly lower value in sarcopenic participants. This is also in line with previous studies [24,32] that have given normative sarcopenic PhA values. Importantly, our study extends these findings by showing that PhA remains informative even when measured using a seated-position BIA device with integrated electrodes. This suggests that despite potential alterations in measurement geometry, PhA retains its clinical relevance and may serve as a biomarker of muscle quality in routine geriatric assessment [25]. Given its non-invasive nature and rapid acquisition, PhA could complement traditional measures of muscle mass and strength in sarcopenia screening strategies [33].

Several limitations should be acknowledged. First, the relatively small sample size, particularly the limited number of sarcopenic participants and male subjects, requires caution in interpreting comparisons between sarcopenic and non-sarcopenic groups. Indeed, the study was not powered for robust age- or sex-stratified subgroup analyses and may reduce statistical power to detect subtle differences in agreement between methods. Larger studies are thus required to confirm the generalizability of our findings across these subgroups. Future studies including larger and more sex-balanced cohorts are therefore needed to confirm these findings and strengthen external validity. Finally, although the integrated electrode design improves the feasibility of seated BIA measurements, electrode–skin contact may be affected by skin characteristics, particularly in older adults. While all assessments were conducted using a standardized protocol and demonstrated good agreement with DXA, the potential influence of skin condition on measurement accuracy was not specifically evaluated. Future studies should investigate the robustness of integrated electrode measurements in populations with greater variability in skin integrity and hydration status. A key strength of this study lies in the use of a seated-position BIA device with integrated electrodes. This configuration was specifically chosen to improve feasibility, efficiency and acceptability in older adults in routine clinical settings. As such, the protocol enhances the ecological validity of the findings. Unlike most previous validation studies performed under standardized supine BIA conditions, the present work evaluates muscle mass estimation in a clinically relevant seated position and directly compares the performance of two commonly used predictive equations (Kyle and Sergi) within the same device. In addition, the study explored whether sarcopenia status influenced measurement validity and examined the clinical relevance of a phase angle derived from seated measurements. Together, these elements provide novel insights into the applicability of seated BIA in older populations.

## 5. Conclusions

The present study demonstrates that a seated-position BIA device with integrated electrodes provides clinically acceptable estimates of ASMM and ASMI in older adults when compared with the reference method DXA. Among the two predictive equations evaluated, the Sergi equation showed superior performance, with higher concordance, lower estimation error, reduced systematic bias, and no evidence of proportional bias. These findings suggest that population-specific equations developed in older adults are better suited for muscle mass assessment in geriatric populations than equations derived from mixed-age cohorts. In addition, PhA measured in a seated position was associated with handgrip strength and was significantly lower in sarcopenic participants, supporting its potential value as an accessible marker of muscle quality and functional status. Taken together, these results indicate that seated-position BIA may represent a feasible and clinically relevant alternative for routine assessment of muscle mass in older adults when DXA is unavailable, impractical, or unsuitable for repeated evaluations, particularly when using the Sergi predictive equation. Further studies are warranted to confirm these findings in larger populations and to evaluate the responsiveness of this approach to longitudinal changes in muscle health.

## Figures and Tables

**Table 1:** EWGSOP2 sarcopenia cut-off points for low strength by chair stand and handgrip strength and for low muscle quantity (ASMM and ASMI).

Tests	Cut-Off Points for Men	Cut-Off Points for Women
Handgrip strength (kg)	<27	<16
5-STS (s)	>15	
ASMM (kg)	<20	<15
ASMI (kg/m^2^)	<7.0	<5.5

5-STS: 5-Sit-To-Stand test; ASMM: Appendicular Skeletal Muscle Mass; ASMI: Appendicular Skeletal Muscle Mass Index.

**Table 2:** Anthropometric and clinical variables (mean ± SD) for the entire population (n = 59), and differences between non-sarcopenic and sarcopenic participants and between males and females.

Variable	Non-Sarcopenic (n = 49)	Sarcopenic (n = 10)	Male (n = 15)	Female (n = 44)	Total (n = 59)
Male/Female (%)	26.53/73.47	20.00/80.00	100/0	0/100	25.42/74.58
Age (years)	73.64 ± 6.73	82.12 ± 5.53	72.03 ± 7.24	76.12 ± 7.04	75.08 ± 7.25 *
Height (cm) ᵃ	164.39 ± 9.07	164.30 ± 9.70	177.27 ± 4.73	159.98 ± 5.15	164.37 ± 9.09 ‡
Weight (kg)	66.55 ± 11.98	60.50 ± 11.28	77.67 ± 8.98	61.39 ± 9.93	65.53 ± 11.99 ‡
BMI (kg/m^2^) ᵃ	24.55 ± 3.48	22.37 ± 3.52	24.78 ± 3.28	23.98 ± 3.66	24.18 ± 3.55
SARC-F ᵃ	0.71 ± 0.91	4.90 ± 3.38	0.60 ± 0.91	1.71 ± 2.47	1.42 ± 2.23 *
handgrip (kg) ᵃ	20.73 ± 8.38	12.98 ± 6.40	30.69 ± 7.95	15.57 ± 4.30	19.42 ± 8.55 * ‡
5-STS time (s)	8.48 ± 1.92	21.20 ± 7.42	9.59 ± 3.63	9.96 ± 5.35	9.87 ± 4.94*
ASMI DEXA (kg/m^2^) ᵃ	6.29 ± 0.92	5.57 ± 0.78	7.09 ± 0.89	5.85 ± 0.72	6.17 ± 0.94 ‡
ASMI sergi BX3 (kg/m^2^)	6.41 ± 0.89	5.90 ± 0.97	7.39 ± 0.60	5.96 ± 0.70	6.32 ± 0.92 ‡
ASMI kyle BX3 (kg/m^2^) ᵃ	6.65 ± 1.08	6.08 ± 1.23	7.97 ± 0.69	6.08 ± 0.78	6.56 ± 1.12 ‡
ASMM DEXA (kg) ᵃ	17.17 ± 3.94	15.22 ± 3.19	22.28 ± 2.80	14.98 ± 1.94	16.84 ± 3.87 ‡
ASMM sergi BX3 (kg) ᵃ	17.52 ± 3.95	16.10 ± 4.11	23.19 ± 1.81	15.26 ± 1.97	17.28 ± 3.97 ‡
ASMM kyle BX3 (kg) ᵃ	18.21 ± 4.65	16.67 ± 4.94	25.01 ± 2.18	15.54 ± 2.18	17.95 ± 4.69 ‡
PhA (°) ᵃ	5.43 ± 0.95	4.32 ± 0.77	5.49 ± 0.71	5.16 ± 1.09	5.24 ± 1.01 *

* (*p* < 0.05): significant difference between non-sarcopenic and sarcopenic participants. ‡ (*p* < 0.05): significant difference between male and female. ᵃ non-parametric test applied when there was deviation from normality (Shapiro–Wilk test, *p* < 0.05 I at least one group). Difference in sex distribution between the two groups (sarcopenic and non-sarcopenic) was compared with a Chi-squared test. BMI: Body Mass Index. ASMM: Appendicular Skeletal Muscle Mass. ASMI: Appendicular Muscle Mass Index; PhA: phase angle at 50 kHz.

**Table 3:** Agreement between BIA-derived estimates (Sergi and Kyle) and DXA for ASMM and ASMI.

Outcome		CCC	Bias	95% LoA	RMSE	MAE	Proportional Bias (*p*-Value)
ASMM	Sergi vs. DXA	0.942	0.44 kg	−2.04 to 2.92 kg	1.33 kg	1.04 kg	*p* = 0.517
	Kyle vs. DXA	0.898	1.11 kg	−2.08 to 4.30 kg	1.96 kg	1.46 kg	*p* < 0.001
ASMI	Sergi vs. DXA	0.865	0.16 kg/m^2^	−0.74 to 1.05 kg/m^2^	0.48 kg/m^2^	0.38 kg/m^2^	*p* = 0.744
	Kyle vs. DXA	0.796	0.39 kg/m^2^	−0.71 to 1.48 kg/m^2^	0.68 kg/m^2^	0.53 kg/m^2^	*p* = 0.009

ASMM: Appendicular Skeletal Muscle Mass; ASMI: Appendicular Skeletal Muscle Mass Index; CCC: Lin’s Concordance Correlation Coefficient; LoA: Limits of Agreement; RMSE: Root Mean Square Error; MAE: Mean Absolute Error. Proportional bias was assessed by linear regression of the differences between methods against their mean values.

## Data Availability

The data generated and/or analyzed during the current study are not publicly available due to ethical and privacy restrictions but may be obtained from the corresponding author upon reasonable request and subject to approval by the relevant ethics committee.
